# Valorisation of Partially Defatted Pistachio Flour as a Value-Added Ingredient for Gluten-Free Cookie Production

**DOI:** 10.3390/foods15213974

**Published:** 2026-11-05

**Authors:** Rito J. Mendoza-Pérez, Elena Álvarez-Olmedo, Ainhoa Vicente, Felicidad Ronda, Pedro A. Caballero

**Affiliations:** 1 Department of Agriculture and Forestry Engineering, Food Technology, College of Agricultural and Forestry Engineering, University of Valladolid, 34004 Palencia, Spain; ritojose.mendoza@uva.es (R.J.M.-P.); elena.alvarez.olmedo@uva.es (E.Á.-O.); mfronda@uva.es (F.R.); 2 PROCEREALtech Group, Research Institute on Bioeconomy—BioEcoUVa, University of Valladolid, 47011 Valladolid, Spain

**Keywords:** pistachio, defatted flour, by-product, cookie, gluten free, rheology, sensory analysis

## Abstract

Valorisation of oil-extraction by-products as food ingredients can improve resource efficiency while enhancing the nutritional quality of gluten-free products. This study evaluated partially defatted pistachio flour (PDPF) as a substitute for rice flour (10–40%) in gluten-free cookies. Dough rheology was characterised by oscillatory and creep–recovery tests, while cookie proximate composition, physical properties and sensory acceptance were determined. PDPF incorporation progressively increased maximum creep compliance from 21 to 36 MPa^−1^ and decreased steady-state viscosity, indicating greater deformability. In the cookies, protein increased from 4.1 to 14.3 g/100 g and fibre from 0.8 to 3.7 g/100 g, while fat remained essentially constant through formulation adjustment. PDPF reduced spread factor and markedly increased breaking force, which rose from 5.0 to 23.8 N, and produced progressively darker and redder cookies. Despite these instrumental changes, consumer texture-liking remained comparable to the control up to 30% PDPF. Cookies containing 10–30% PDPF showed higher appearance, taste and overall acceptance than the rice-flour control, whereas the 40% formulation lost this sensory advantage. A 30% PDPF substitution provided the most favourable balance between nutritional enrichment, technological performance and consumer acceptance, supporting the use of PDPF as a high-value ingredient for gluten-free cookie production and contributing to pistachio by-product valorisation.

## 1. Introduction

The food industry generates substantial amounts of waste and by-products during the processing of plant-based raw materials, including cereals, pseudocereals, legumes, fruits, vegetables, oilseeds, and nuts. These materials are frequently underutilised or discarded, despite often retaining valuable nutritional and bioactive compounds that could be recovered and incorporated into new food products [1]. Their valorisation therefore represents an attractive strategy for reducing food waste, improving resource efficiency, and promoting a circular economy within the agri-food sector [2]. In parallel, the growing demand for healthier and more sustainable foods has increased interest in the development of value-added ingredients from plant-based agri-food by-products [3,4]. In this context, nuts are of particular interest among plant-based raw materials because of their high nutritional value [5] and the increasing scale of production, which reached 18.4 million tonnes in 2024, with pistachio being among the most relevant nut crops [6]. However, the growing production and diversification of pistachio-derived products have also increased the generation of processing by-products, including shells, hulls, stems, leaves, and residues from oil extraction [7,8,9].

The high lipid content of pistachio kernels and the favourable profile of their fatty acids make pistachio an attractive raw material for oil extraction. Pistachio oil production may also provide an alternative route for the utilisation of kernels that do not meet the quality standards required for direct consumption, including smaller, broken, closed, or immature nuts [10]. Oil extraction generates a totally or partially defatted by-product, commonly referred to as an oil cake or press cake, which is of particular interest for food applications because it retains substantial amounts of valuable nutrients, including proteins, fibre, residual lipids, vitamins, minerals, and bioactive compounds [11,12]. Salinas et al. [13] reported that the by-product obtained after partial pistachio oil extraction contained 33.2% protein, 30.6% residual fat, 13.6% total dietary fibre, and 3.5% ash. The residual lipid fraction was mainly composed of mono- and polyunsaturated fatty acids, while the mineral fraction included relevant amounts of potassium, phosphorus, magnesium, and calcium. Furthermore, the techno-functional properties reported for pistachio oil-extraction by-products support their potential use as high-value ingredients in the formulation of new foods [10,12,13].

Despite their nutritional and techno-functional potential, partially defatted cakes from seeds and nuts have traditionally been used mainly for animal nutrition, while other applications include their use as dietary supplements, fertilisers, substrates for biogas generation, and raw materials for biotechnological processes such as the production of industrial enzymes, vitamins, and other biochemical compounds [14]. More recently, interest in their potential application for human consumption has grown. Partially defatted cakes from seeds and nuts can be easily transformed into flour through a milling process, providing a particle size suitable for use in bakery and pastry products. Their incorporation into the qualitative formulation of these foods has been proposed as a strategy to improve their nutritional profile [10,15]. Numerous recent studies have focused on the development of bakery and pastry products using flours derived from partially defatted cakes of various oilseed species and nuts, such as hemp [16], sesame [2,17], sunflower [2], peanut [18], walnuts [19], and chia, flax, sesame, and poppy seeds [20]. These studies demonstrate the potential of partially defatted flours as ingredients for the development of bakery products with modified nutritional, technological, and sensory properties.

The use of these ingredients may be particularly relevant in gluten-free (GF) products. GF bakery formulations are commonly based on cereal flours and starches that lack the structural contribution of gluten and may also present nutritional limitations, particularly in terms of protein and dietary fibre [21]. Consequently, the incorporation of partially defatted oilseed and nut flours has been proposed as a strategy to improve the nutritional profile of GF baked products while also modifying their technological and sensory properties [19,22]. Nevertheless, studies addressing the incorporation of this type of flour into GF bakery products, particularly cookies, remain relatively scarce and have mainly focused on a limited number of oilseed and nut species. Radočaj et al. [16] evaluated the impact of replacing rice flour with partially defatted hemp flour in GF cracker formulations, obtaining the highest sensory rating for the product when a substitution percentage of 20% (*w*/*w*) was used. Martínez et al. [20] analysed the influence of defatted flours from chia, flaxseed, sesame, and poppy seeds on the physical, nutritional, and sensory properties of GF cookies and compared them with an equivalent wheat-based product. In the case of nuts, Martendal et al. [19] developed GF cookies based on a formulation containing rice flour and corn starch, employing partially defatted pecan flour at substitution levels of 15% and 30% (*w*/*w*), which demonstrated an improvement in product texture. These findings highlight the potential of partially defatted seed and nut flours for GF bakery applications, although their effects depend strongly on the composition and functional characteristics of the specific raw material used.

Regarding pistachio-derived ingredients, partially defatted pistachio flour (PDPF) has been previously investigated in wheat-based breadmaking. Guardianelli et al. [7] evaluated the effect of partially replacing wheat flour with PDPF on the rheological, thermal, and hydration properties of the resulting doughs. These results demonstrated that PDPF can markedly affect dough structure and functionality. However, wheat-based systems differ substantially from GF formulations because gluten plays a central role in dough structure and processing behaviour. Therefore, the effects observed in wheat doughs cannot be directly extrapolated to GF systems. In rice-based GF doughs, the replacement of starch-rich flour with PDPF increases the relative contribution of proteins and fibre, potentially altering water distribution and interactions among dough components, while residual pistachio lipids may further influence the resulting structure and rheological behaviour. To the best of our knowledge, the use of PDPF obtained as a by-product of pistachio oil extraction as an ingredient in GF bakery products has not yet been investigated. Considering its high contents of protein, fibre, residual lipids, minerals, and other valuable compounds, its incorporation could provide a suitable approach for simultaneously valorising a pistachio-processing by-product and developing GF products with modified nutritional and technological characteristics.

We hypothesised that the partial replacement of rice flour with PDPF would modify dough rheology and cookie characteristics due to changes in composition and interactions within the dough matrix. Accordingly, the aim of the present study was to evaluate the effect of partially replacing rice flour with PDPF at increasing substitution levels (10%, 20%, 30%, and 40%) in a gluten-free cookie formulation. First, the rheological behaviour of the resulting doughs was characterised by dynamic oscillatory and creep-recovery tests to determine the changes in their viscoelastic and deformation properties induced by PDPF incorporation. The resulting cookies were evaluated in terms of proximate composition, morphogeometric properties, texture, and colour, and their sensory characteristics and overall acceptance were assessed through a consumer hedonic test.

## 2. Materials and Methods

### 2.1. Raw Materials

The pistachios used in this study were natural kernels without shells (4.1 ± 0.2% moisture, 46 ± 1% fat, 9 ± 2% fibre, 26 ± 2% protein, and 3.0 ± 0.4% ash), sourced from smaller nuts or those of lower quality, such as broken, closed, or immature nuts, provided by Pistacyl (Valladolid, Spain). These pistachios were stored at 10 ± 2 °C prior to processing and analysis. Partially defatted pistachio flour (PDPF) was obtained as a by-product of oil extraction from pre-treated natural pistachio kernels sliced to a thickness of 1.5 mm and pretreated at 25 °C for 30 min. Oil was extracted using a single-screw press (CZR 309, Goldenwall, Hangzhou, China), following the procedures described by Mendoza-Pérez et al. [10]. The resulting press cake was ground using an electric grinder (HC-400, Cgoldenwall, Hangzhou, China) and sieved through a 500 µm mesh to obtain PDPF. The obtained PDPF was preserved at 10 ± 2 °C until use. The proximate composition of PDPF, expressed as % (*w*/*w*), was as follows: 3.8 ± 0.1% moisture, 16 ± 1% fat, 12 ± 2% fibre, 44 ± 3% protein, and 6 ± 0.4% ash. The moisture, protein, fat, and ash contents of pistachio kernels and their derivatives were determined according to the AACC Official Methods 44-19.01, 46-10.01 (conversion factor 6.25), 30-25.01, and 08-01.01, respectively [23]. The fibre content was analysed following AOAC Method 991.43 [24]. Total carbohydrate content was calculated by difference. Analyses were performed in duplicate. The particle size distribution of PDPF, determined by sieving, was 24% between 355 and 500 μm, 25% between 250 and 355 μm, 24% between 180 and 250 μm, 24% between 106 and 180 μm, and 3% below 106 μm.

The micronized rice flour kindly supplied by Herba Ricemills S.L.U (Tarragona, Spain) was used in this study to prepare GF cookies. The moisture content was 12.5%, fat 0.5%, protein 6.5%, and ash 0.9%. The flour particle size distribution was 1% > 150 μm, 5–15% between 150 and 90 μm, and 84% < 90 μm (data provided by the manufacturer).

Sugar, butter, sodium bicarbonate, salt, and vanilla extract were purchased from the local market. Water from the local water supply was used for dough preparation.

### 2.2. Dough Preparation and Cookie-Making

The GF cookie formulation was adapted from the recipe reported by Hu et al. [25], with modifications developed through preliminary tests. The formulation is presented in Table 1.

For the PDPF-containing formulations, rice flour was partially replaced with PDPF at nominal substitution levels of 10%, 20%, 30%, and 40%. The formulations were designated as P0 for the control containing 100% rice flour and P10, P20, P30, and P40 for the formulations containing 10%, 20%, 30%, and 40% PDPF, respectively, in the flour mixture. These nominal substitution levels were established relative to the initial rice flour content of the control formulation, before adjusting the ingredient quantities for fat and moisture.

Because PDPF contained a higher amount of fat than rice flour, the amount of added butter was reduced in each formulation to compensate for the fat supplied by PDPF and thus maintain a constant total fat content across formulations. The amount of added water was also adjusted according to the actual moisture content of each flour mixture, using a flour moisture content of 13% as the reference, and to butter moisture (Table 1). All remaining ingredients were maintained at the same levels as in the control formulation.

Five different cookie dough formulations were prepared by first mixing the butter with sugar using a KitchenAid Professional 5KPM50 mixer (KitchenAid, St. Joseph, MI, USA) for 3 min. Subsequently, the liquid ingredients were added to the mixture, followed by the incorporation of the flour or flour mixtures, along with the remaining ingredients. The dough was allowed to rest at 20 ± 2 °C for 30 min, and was rolled out using a SALVA L-500-J laminator (Salva Industrial S.L.U., Lezo, Spain) adjusted to a roller gap of 6 mm. The cookies were cut using a circular mould with an internal diameter of 40 mm and baked in a Sveba Dahlen oven (Sveba Dahlen AB, Fristad, Sweden) at 180 °C for 11 min. Two batches were prepared for each formulation. The cookies were then left to cool for one hour at a controlled room temperature (20 ± 2 °C) before their physical properties were analysed.

### 2.3. Rheological Characterisation of Doughs

Cookie dough rheology was carried out with a Kinexus Pro + rheometer (Malvern Instruments Ltd., Malvern, UK) with parallel plate geometry (40 mm diameter) of serrated surface and with 4 mm gap. The excess of dough was removed, and the exposed sample surfaces were covered with paraffin oil to prevent moisture loss. Measurements were started 10 min after dough preparation. For each test, the sample was positioned between the plates and allowed to relax for 5 min at a controlled temperature of 25 °C using a Peltier plate. Oscillatory rheological measurements were performed in duplicate, while creep–recovery tests were performed in triplicate.

Strain sweep tests were performed within the strain range of 0.01% to 200% at a constant frequency of 1 Hz. The linear viscoelastic region (LVR) was determined by identifying the maximum stress (τ_max_) that the doughs could withstand before suffering irreversible deformation, which marks the end of the LVR. The crossover point was also determined as the stress where the elastic modulus was equal to the viscous modulus (G′ = G″) [26,27].

Frequency sweeps were conducted within the range of 10 to 1 Hz at a strain of 0.02%. The elastic (G′) and viscous (G″) moduli, along with the loss tangent (tanδ), were obtained from the frequency sweeps and subsequently fitted to the power law model, as previously employed by Ronda et al. [27]. The coefficients G_1_′, G_1_″, and tan(δ)_1_, obtained from the power law fittings, represent the elastic and viscous moduli and the loss tangent at a frequency of 1 Hz, respectively. The exponents *a*, *b*, and *c* quantify their dependence on the oscillation frequency.

Creep-recovery tests were conducted by applying a constant stress of 100 Pa, within the LVR, and maintaining this stress for 60 s. Subsequently, the stress was removed, and the recovery phase was recorded for an additional 180 s. The data obtained during the creep phase were fitted to the four-parameter Burgers model (Equation (1)), whereas the recovery phase was fitted independently to the corresponding three-parameter Burgers model (Equation (2)), in which the viscous flow component is omitted because no viscous flow occurs during recovery, following previous studies [26,27].

(1) $$J_{c}\left(t\right)=J_{0c}+J_{1c}\left(1-\exp\left(\frac{-t}{\lambda_{1c}}\right)\right)+\frac{t}{{µ}_{0}}$$

(2) $$J_{r}\left(t\right)=J_{\max}-J_{0r}-J_{1r}\left(1-\exp\left(\frac{-t}{\lambda_{1r}}\right)\right)$$

The coefficients J_c_(t) and J_r_(t) represent the creep and recovery compliances (strain divided by stress), respectively. J_0_ is the instantaneous compliance, J_1_ is the retarded elastic compliance, λ_1_ is the retardation time, and µ_0_ is the steady-state viscosity during the creep phase. The subscript “c” denotes parameters obtained from the creep phase, whereas the subscript “r” denotes those obtained from the recovery phase. For the recovery phase, time was defined from the moment of stress removal (t_r_ = 0 at 60 s of the experiment). J_max_ is the maximum creep compliance reached at the end of the creep step. The expression [(J_0c_ + J_1c_)/J_max_] represents the ratio between elastic compliance (instantaneous + retarded) and total compliance (elastic + viscous) [28].

### 2.4. Proximate Composition of Cookies

The moisture, protein, fat and ash content of the cookies were determined in duplicate according to the AACC official methods 44-19.01, 46-13.01 (N × 6.25), 30-10.01 and 08-01.01, respectively [23]. The AOAC method 991.43 [24] was used to determine the dietary fibre of the samples. Their carbohydrate content was determined by difference, whereby the sum of the measured percentages of moisture, protein, fat, fibre, and ash was subtracted from 100. The energy value was estimated from the mean content of protein, fat, fibre, and carbohydrates, using the energy conversion factors specified in Regulation (EU) No 1169/2011, 4.0, 9.0, 2.0 and 4.0 kcal/g for each component, respectively [29].

### 2.5. Physical Properties of Cookies

#### 2.5.1. Morphogeometric Properties of Cookies

The morphogeometric properties of the cookies were determined based on their diameter and thickness. These parameters were measured on 10 baked cookies of each formulation using a digital calliper. The spread factor was then calculated by dividing the cookie’s diameter by its thickness.

#### 2.5.2. Texture Properties of Cookies

The texture of GF cookies was evaluated using a TA-XT2 texture analyser (Stable Microsystems, Godalming, UK), equipped with the Exponent software (version 6.1.18.0), following the “Three-point bending test” method [30], with slight modifications. A steel HDP/BS blade probe (“Guillotine” blade set) was employed together with a support base consisting of two rounded-end supports separated by 30 mm. The tests were carried out 1 h after baking. The experimental conditions were as follows: trigger force of 0.05 N, probe displacement distance of 10.0 mm, pre-test speed of 1.00 mm/s, test speed of 1.00 mm/s, and post-test speed of 10.00 mm/s. The maximum breaking force (N), corresponding to the highest force peak recorded during the test, was used as a measure of cookie hardness. Five cookies from each independent batch and formulation were analysed.

#### 2.5.3. Colour of Cookies

The colour of the cookies was measured using a colorimeter (PCE-CSM5, PCE Instruments, Meschede, Germany) and CQCS3 software (version 3.2). The CIE L*a*b* coordinates were obtained using a D65 standard illuminant and a 10° standard observer. The lightness (L*) (from 0–black to 100–white) and chromatic coordinates a* (from green (−) to red (+)) and b* (from blue (−) to yellow (+)) were measured. Hue (h) and chroma (C*) were calculated from the CIEL*a*b* coordinates. The colour difference (ΔE) between each cookie sample enriched with PDPF and the control cookie (P0) was calculated using Equation (3):

(3) $$\Delta E={\left[{\left({\Delta L}^{*}\right)}^{2}+{\left(\Delta a^{*}\right)}^{2}+{\left(\Delta b^{*}\right)}^{2}\right]}^{1/2}$$

Five cookies from each batch and formulation were measured at three different points.

### 2.6. Sensory Attributes of Cookies

The sensory evaluation of the cookies was conducted using a hedonic test, with 76 volunteers aged between 18 and 65 years, recruited from the University of Valladolid community (Spain). Regarding demographic and consumption information, approximately 54% of participants were female, 44% male, and 2% identified as other. The age distribution was 32% aged 18–24 years, 41% aged 25–34 years, 13% aged 35–44 years, 10% aged 45–54 years, and 3% aged 55–64 years. Regarding habitual consumption, 75% reported regularly consuming cookies, 37% pistachios, and 15% gluten-free products. Individuals with nut allergies or coeliac disease were not allowed to participate in accordance with the conditions established by the study’s ethics committee approval. The cookies were stored in polyethylene zip-lock bags at a controlled room temperature (20 ± 2 °C) and evaluated one day after baking. The cookies were evaluated based on their appearance, smell, texture, taste, and overall acceptance, using a 9-point hedonic scale ranging from 1 (extremely dislike) to 9 (extremely like). Samples were presented in a randomised order and coded with three-digit random numbers. Water was provided between samples to cleanse the palate.

### 2.7. Statistical Analysis

Statistical analyses were conducted using the Statgraphics Centurion 19 software (Bitstream, Cambridge, MN, USA). Dough and cookie data were analysed using multifactorial analysis of variance (ANOVA), with formulation and production batch as factors. No significant effect of batch was detected for the evaluated parameters (*p* > 0.05). Sensory data were analysed using a one-way repeated-measures ANOVA, with formulation as the within-subject factor and consumer as the subject. Differences among formulations were assessed using Fisher’s least significant difference (LSD) test (*p* < 0.05). Pearson correlation analysis was performed using the mean values of the five formulations to assess linear relationships among the measured variables. Dough and cookie results are reported as means of all measurements obtained from both production batches (with the number of measurements per batch specified for each parameter), together with the pooled standard error (SE) derived from ANOVA.

## 3. Results and Discussion

### 3.1. Rheological Properties of the Doughs

#### 3.1.1. Dynamic Oscillatory Tests

**Figure 1:**
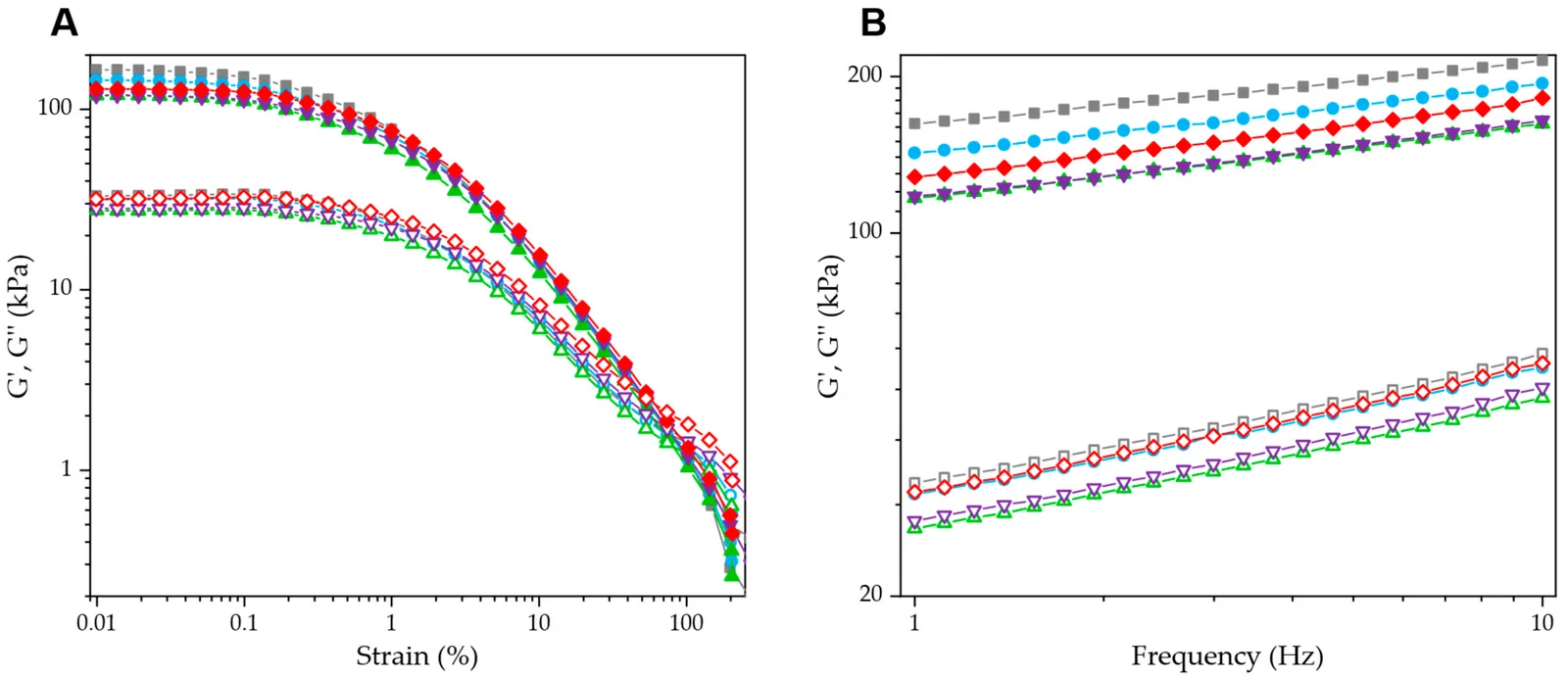
(**A**) Strain sweeps and (**B**) frequency sweeps of doughs made with rice flour and with different levels of substitution with partially defatted pistachio flour. Symbols and colour assignment: Control dough with 100% rice flour: (■) connected with black lines; doughs made with different levels of rice flour substituted with pistachio flour [10% (P10), 20% (P20), 30% (P30), and 40% (P40)]: ●P10, ▲P20, ▼P30 and ♦P40. In sweeps (**A**,**B**), the elastic modulus G′ is represented by solid symbols, and the viscous modulus G″ by empty symbols.

The rheological properties of doughs made with 100% rice flour (control) and with increasing levels of PDPF (10%, 20%, 30%, and 40%) were characterised by dynamic oscillatory tests. The strain and frequency sweep curves obtained for the different doughs are presented in Figure 1A and Figure 1B, respectively, while the corresponding rheological parameters are summarised in Table 2. All doughs exhibited viscoelastic behaviour, with G′ remaining higher than G″ throughout the analysed range, indicating a predominantly elastic solid-like character [27].

The strain sweep tests showed two distinct regions in all doughs: the linear viscoelastic region (LVR) and the nonlinear region. Within the LVR, both G′ and G″ remained relatively constant with increasing strain, indicating that the dough structure was preserved. The maximum stress within the LVR (τ_max_) ranged from 0.17 to 0.22 kPa. P20 showed a significantly lower τ_max_ than P0, P10, and P40, whereas P30 presented an intermediate value. At higher strains, in the nonlinear region, both moduli progressively decreased, with G′ showing a greater decrease than G″ until the crossover point (G′ = G″) was reached, indicating the transition from predominantly elastic to predominantly viscous behaviour. The crossover stress remained practically unchanged among formulations, ranging from 1.7 to 1.8 kPa. Although minimal or non-statistically significant differences were obtained in the calculated parameters derived from strain sweep tests, the doughs containing PDPF generally exhibited slightly lower viscoelastic moduli than the control dough made exclusively with rice flour (P0), with no clear trend observed with increasing PDPF content.

The frequency sweep was conducted at a strain of 0.02%, within the LVR. Frequency sweep data were fitted to the power law model, and the resulting coefficients are summarised in Table 2. The fitted elastic modulus (G_1_′) was approximately 4–5 times higher than the fitted viscous modulus (G_1_″) in all formulations, confirming the predominantly elastic behaviour characteristic of the GF doughs studied [26]. P0 and P10 showed similar G_1_′ values, whereas substitution levels of 20% or higher resulted in significantly lower values, corresponding to reductions of approximately 26–28% relative to the control. A similar, although less pronounced, behaviour was observed for G_1_″, which was also significantly lower in P20, P30, and P40 than in P0 and P10. The loss tangent, tan(δ)_1_, progressively increased from 0.203 in P0 to 0.247 in P40, indicating an increasing viscous contribution relative to the elastic component as PDPF incorporation increased. Previous studies have attributed an increase in tan(δ)_1_ values to a weakening of the viscoelastic structure of dough systems [31]. In the present study, tan(δ)_1_ was strongly positively correlated with the protein (r = 0.988, *p* < 0.01) and fibre contents (r = 0.986, *p* < 0.01), and negatively correlated with carbohydrate content (r = −0.988, *p* < 0.01). The strong associations of tan(δ)_1_ with protein and fibre contents suggest that the progressive change in dough viscoelasticity was related to the compositional changes induced by PDPF incorporation. Similar effects have been reported in gluten-free systems, where partial replacement of starch with rapeseed protein resulted in lower G′ and G″ values and an increased tan(δ), indicating a greater relative contribution of the viscous component [31]. However, the rheological effect of protein- and fibre-rich ingredients depends strongly on their nature and interactions with the starch matrix and water. Villanueva et al. [32] related differences in the viscoelastic response of gluten-free doughs containing tef flour to variations in protein, fibre, and fat contents and their influence on water absorption, while Ronda et al. [33] highlighted the key role of water competition in determining the rheological behaviour of fibre-enriched rice-based doughs.

Interestingly, Guardianelli et al. [7] reported an increase in elastic modulus when pistachio oil-industry by-product flour was incorporated into wheat dough, highlighting the strong influence of the food matrix on the rheological effect of this ingredient.

The parameters *a*, *b*, and *c* quantify the variation in G′, G″, and tan(δ) with respect to the oscillation frequency, respectively, and provide information on the frequency-dependent response of the dough structure [34]. In all doughs, exponent *a* was lower than exponent *b*, resulting in positive values of exponent *c* and, consequently, an increase in tan(δ) with increasing oscillation frequency. Thus, the viscous contribution relative to the elastic contribution increased as frequency increased in all formulations. Regarding the effect of PDPF incorporation, exponent *a* increased significantly at substitution levels of 20% or higher, indicating a greater frequency dependence of the elastic modulus. In contrast, exponent *b* showed only minor variations; none of the PDPF-containing formulations differed significantly from P0, although P30 showed a higher value than P10. Consequently, c was significantly lower in all PDPF-containing doughs than in P0. The increase in the frequency dependence of G′ with increasing PDPF content may indicate a less stable viscoelastic structure [32]. The increase in parameter *a* was also strongly associated with the compositional changes produced by PDPF incorporation, showing positive correlations with protein and fibre contents (r = 0.977 for both, *p* < 0.01) and a negative correlation with carbohydrate content (r = −0.977, *p* < 0.01).

Similar reductions in G′ and G″ have been reported when nut flours were incorporated into wheat dough. Pycia & Juszczak [35] observed progressive decreases in both moduli with increasing hazelnut or walnut flour incorporation, while G′ remained higher than G″ in all formulations. The authors attributed this behaviour mainly to the increased lipid content and dilution of the gluten network. In the present GF system, however, the total fat content of the formulations was adjusted, although the contribution of butter fat decreased as that of residual pistachio oil increased. Replacing starch-rich rice flour with PDPF reduced the relative starch fraction and increased the proportion of protein- and fibre-rich particles, potentially modifying component interactions and competition for available water. These changes may have weakened the elastic contribution to dough structure, consistent with the greater decrease observed in G′ and G″ and the increase in tan(δ). A comparable effect on structural stability has also been reported in a GF system containing partially defatted walnut flour. Burbano et al. [36] observed progressive reductions in critical strain and cohesive energy as walnut flour incorporation increased, indicating a greater susceptibility of the batter structure to deformation.

#### 3.1.2. Creep-Recovery Tests

Rheological measurements within the LVR are useful for evaluating the contribution of dough structural components, owing to the sensitivity of rheological parameters to changes in the type and concentration of the polymers present in the system [33]. While creep-recovery tests performed outside the LVR can provide information on the large deformations experienced by doughs during processing, measurements within the LVR can be more readily related to the molecular structure and interactions of the sample components [28].

**Figure 2:**
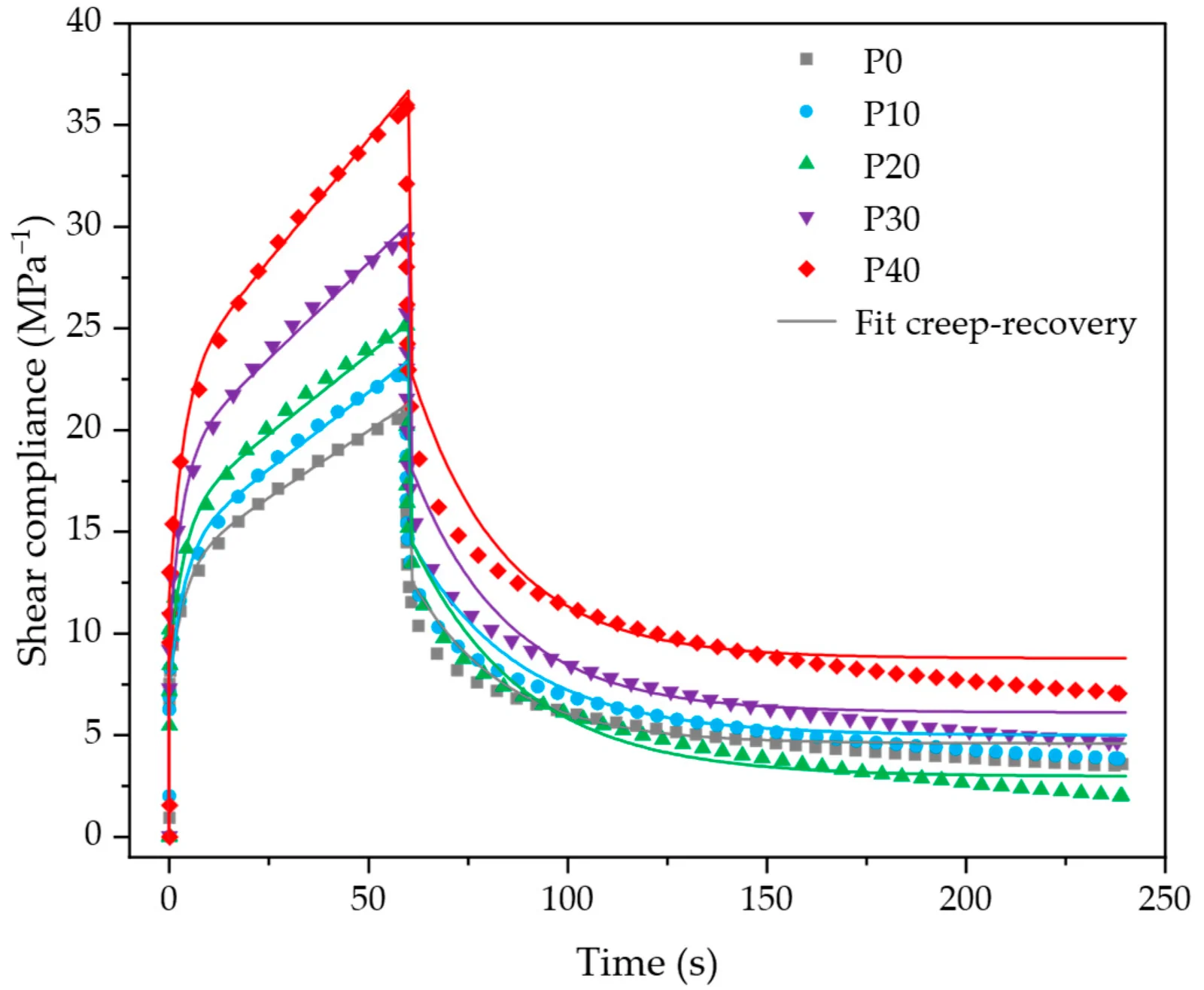
Creep-recovery tests on doughs made with rice flour and with different levels of substitution with partially defatted pistachio flour. Experimental data are represented by filled symbols. Solid lines represent the curves calculated from the mean Burgers model parameters (Table 2) for the creep (0–60 s) and recovery (60–240 s) phases, respectively.

Figure 2 shows the creep-recovery curves obtained for the studied doughs. A constant stress of 100 Pa, within the LVR, was applied for 60 s during the creep phase, followed by a 180 s recovery period after stress removal. All formulations exhibited the characteristic viscoelastic response of dough systems, combining instantaneous and time-dependent deformation during creep with partial recovery after stress removal, as previously reported for rice flour doughs [37] and other GF doughs [26,27,33,38].

The parameters obtained by fitting the creep and recovery curves to the Burgers models are presented in Table 2. Both the instantaneous compliance (J_0_) and the retarded elastic compliance (J_1_) progressively increased with increasing PDPF incorporation during both the creep (c) and recovery (r) phases. The largest changes were observed in P40, with increases relative to P0 of 57% for J_0c_ and 80% for J_1c_ during creep, and 60% for J_0r_ and 75% for J_1r_ during recovery. Similarly, J_max_ increased from 21 MPa^−1^ in P0 to 36 MPa^−1^ in P40, with substitution levels of 20% or higher showing significantly higher values than the control. Since compliance represents the deformation produced by a given applied stress, these results indicate that PDPF incorporation progressively increased dough deformability and reduced its resistance to deformation [39]. This behaviour was strongly associated with the compositional changes induced by PDPF incorporation. J_0c_, J_1c_, J_max_, J_0r_, and J_1r_ showed significant positive correlations with protein and fibre contents (r = 0.948 to 0.989, *p* < 0.05) and negative correlations with carbohydrate content (r = −0.948 to −0.988, *p* < 0.05). Conversely, steady-state viscosity (μ_0_) was negatively correlated with both protein and fibre contents (r = −0.981 for both, *p* < 0.01) and positively correlated with carbohydrate content (r = 0.982, *p* < 0.01). These associations support the relationship between the progressive replacement of the starch-rich rice flour fraction by protein- and fibre-rich PDPF and the increased deformability of the resulting doughs. However, because protein, fibre, and carbohydrate contents changed simultaneously with PDPF substitution, their individual contributions to the rheological response cannot be separated from these correlations.

The retardation time during creep (λ_1c_) was not significantly affected by PDPF incorporation. During recovery, λ_1r_ showed only limited differences, with P20 presenting a higher value than P0 and P40, whereas P10 and P30 showed intermediate values. However, the retardation times obtained during recovery were considerably higher than those obtained during creep. The recovery fits showed lower R^2^ values (0.953–0.970 versus 0.993–0.995 for creep). Given the comparatively lower goodness of fit during recovery, the parameters derived from this phase should be interpreted with greater caution.

μ_0_ progressively decreased from 7.6 MPa·s in P0 to 4.2 MPa·s in P40, with significantly lower values observed for P30 and P40 compared with the control, further indicating a lower resistance to flow as PDPF incorporation increased. Despite these marked changes in absolute compliance and viscosity, the relative contribution of the elastic component, expressed as [(J_0c_ + J_1c_)/J_max_], remained unchanged among formulations. Thus, PDPF incorporation increased dough deformability and reduced resistance to viscous flow without significantly altering the relative elastic contribution estimated from the creep response. The internal relationships among the creep parameters further supported this response. J_0c_ was strongly positively correlated with J_1c_ (r = 0.98, *p* < 0.01), while μ_0_ decreased with increasing J_0c_ (r = −0.96, *p* < 0.05) and J_1c_ (r = −0.99, *p* < 0.001).

The increased susceptibility to deformation observed in the present study is consistent with the behaviour reported by Pycia & Juszczak [35] for wheat doughs containing hazelnut or walnut flour. Their creep-recovery analysis showed that nut-flour-containing doughs were more susceptible to deformation than the control, an effect attributed primarily to disruption and dilution of the gluten network. This mechanism does not apply to the present GF doughs, which lack the gluten network that provides much of the elasticity in wheat doughs. Instead, their viscoelastic behaviour depends on the starch-rich matrix and interactions among starch, proteins, fibre, lipids, and water, which may have been modified by PDPF incorporation, contributing to the increased compliance. In GF systems, the effect of added protein is also strongly dependent on the surrounding starch matrix and its interactions with other formulation components. Villanueva et al. [28], for example, showed that protein fortification modified the rheological response of starch-based GF doughs to different extents depending on the starch source.

### 3.2. Proximate Composition of Cookies

The incorporation of partially defatted pistachio flour (PDPF) significantly modified the proximate composition of the GF cookies (Table 3). Protein content increased progressively with PDPF substitution, from 4.1 g/100 g in P0 to 6.6, 9.2, 11.7, and 14.3 g/100 g in P10, P20, P30, and P40, respectively, with significant differences among all formulations (*p* < 0.05). This increase reflects the high protein content of PDPF compared with rice flour and demonstrates its potential to improve the nutritional profile of GF bakery products.

Dietary fibre content also increased with PDPF incorporation, from 0.8 g/100 g in P0 to 3.7 g/100 g in P40. Although P10 did not differ significantly from the control, fibre contents were significantly higher at substitution levels of 20% or above. Ash content followed a similar progressive increase, from 0.60 g/100 g in P0 to 1.98 g/100 g in P40, consistent with the higher mineral content of PDPF. Conversely, carbohydrate content decreased significantly and progressively from 71 g/100 g in P0 to 55 g/100 g in P40 as the starch-rich rice flour fraction was replaced by PDPF.

Fat content remained practically constant across formulations (approximately 23 g/100 g), confirming the effectiveness of adjusting the amount of added butter to compensate for the residual fat supplied by PDPF. However, maintaining a similar total fat content did not ensure an unchanged fatty acid profile, since the contribution of butter fat, which is relatively rich in saturated fatty acids, progressively decreased, whereas that of residual pistachio oil, generally rich in unsaturated fatty acids, increased [10]. These concurrent changes may also have influenced dough behaviour and cookie properties, but their individual contributions cannot be distinguished from those of the other compositional changes. This aspect differentiates the present formulation strategy from previous studies using defatted seed or nut flours, in which incorporation of the oil-extraction by-product also resulted in increased fat contents. Martínez et al. [20], for example, reported higher protein and fibre contents and lower carbohydrate contents in cookies prepared with defatted flax, sesame, chia, and poppy seed flours, but also observed increases in fat content associated with the residual oil present in these ingredients. Similarly, Martendal et al. [19] reported increases in protein, insoluble fibre, and lipid contents and a reduction in carbohydrates when pecan press-cake flour was incorporated into GF cookies.

PDPF-containing cookies also showed slightly higher moisture contents (1.2–1.6 g/100 g) than P0 (0.6 g/100 g), although no progressive effect of substitution level was observed. Despite these compositional changes, all cookies showed very low moisture contents. The calculated energy value decreased slightly with PDPF incorporation, from 509 kcal/100 g in P0 to 493 kcal/100 g in P40. This limited reduction is consistent with the maintenance of a constant fat content across formulations, while part of the carbohydrate fraction was progressively replaced by protein and fibre.

### 3.3. Physical Properties of Cookies

Table 4 summarises the physical properties of cookies prepared with rice flour and increasing levels of PDPF, including spread factor, maximum breaking force, and colour parameters.

#### 3.3.1. Morphogeometric Analysis

The spread factor is an important indicator of cookie geometry and reflects the ability of the dough to spread during baking, which is influenced by formulation, dough consistency, and water availability [40]. PDPF incorporation significantly reduced the spread factor from 5.1 in P0 to 4.7–4.8 in the PDPF-containing cookies. However, no significant differences were observed among P10, P20, P30, and P40, indicating that the reduction occurred after PDPF incorporation but did not increase further with substitution level. Cookie diameter did not differ significantly among formulations, whereas thickness increased significantly by approximately 6–9% in PDPF-containing cookies compared with P0, without a consistent trend with substitution level. Therefore, the reduction in spread factor was mainly associated with increased thickness rather than changes in diameter.

Changes in cookie spread associated with flour substitution can arise from differences in protein and fibre contents and their effects on dough hydration and consistency [41]. In particular, Mancebo et al. [42] reported that insoluble fibres increased dough consistency and produced cookies with a lower spread factor, whereas soluble fibres showed the opposite behaviour. This effect has been associated with the water-binding capacity of fibre-rich ingredients, which reduces the amount of free water available in the dough, increases dough consistency, and consequently limits cookie spread during baking. Therefore, the higher protein and fibre contents of PDPF compared with rice flour, together with the associated changes in interactions with water and other dough components, may have contributed to the lower spread observed in the PDPF-containing cookies.

#### 3.3.2. Texture Analysis

Textural properties are among the main factors determining cookie quality and consumer perception. The maximum breaking force obtained from the three-point bending test was used as an indicator of cookie hardness [43,44]. The inclusion of increasing levels of PDPF resulted in a significant and progressive increase in maximum breaking force, from 5.0 N in P0 to 6.6, 11.1, 22.1, and 23.8 N in P10, P20, P30, and P40, respectively. These values corresponded to increases of 32%, 122%, 342%, and 376% relative to the control, with significant differences among all formulations. Although the slight increase in cookie thickness (6–9%) may have influenced breaking force, changes in thickness did not parallel the increase in breaking force. Notably, P10 and P40 did not differ significantly in thickness, whereas breaking force increased markedly from 6.6 to 23.8 N, suggesting that cookie geometry alone cannot explain the observed hardening.

The increase in cookie hardness was strongly associated with the compositional changes induced by PDPF incorporation. Maximum breaking force was positively correlated with protein (r = 0.96, *p* < 0.01) and fibre contents (r = 0.97, *p* < 0.01), and negatively correlated with carbohydrate content (r = −0.96, *p* < 0.01). These relationships suggest that the progressive replacement of starch-rich rice flour by protein- and fibre-rich PDPF was closely associated with the development of a more resistant structure after baking.

Cookie hardness was also strongly associated with the rheological behaviour of the corresponding doughs. Maximum breaking force correlated positively with J_1c_ (r = 0.97, *p* < 0.01) and J_0r_ (r = 0.96, *p* < 0.01), and negatively with μ_0_ (r = −0.95, *p* < 0.05). Significant positive correlations were also observed with J_0c_ and J_max_ (r ≈ 0.94, *p* < 0.05). Thus, formulations producing more deformable doughs and exhibiting lower resistance to flow before baking subsequently produced harder cookies.

The compositional characteristics of PDPF provide several possible explanations for this behaviour. Protein enrichment can alter starch–protein interactions and the structure developed during baking. Previous studies have shown that changes in protein source and concentration can markedly modify the texture of GF cookies, although the direction of the effect depends on the flour matrix and the interactions established among proteins, starch, and other formulation components [45,46]. Altındağ et al. [46], for example, reported significant effects of flour composition and protein content on the hardness of GF cookies prepared from buckwheat, corn, and rice flours.

Fibre may also contribute substantially to the observed changes in texture, but its effect depends on its amount and physicochemical characteristics. Laguna et al. [47] demonstrated that fibre morphology strongly affected biscuit texture: wheat fibres increased resistance to breaking, whereas apple fibre produced a crumblier structure. Similarly, Martendal et al. [19] reported a markedly higher cutting force in GF cookies containing pecan press-cake flour and attributed this mainly to their higher insoluble fibre content. Pineli et al. [48] found that hardness and fracturability increased at high replacement levels of partially defatted baru flour, an oil-extraction by-product rich in protein and fibre. These findings are consistent with the increased breaking force observed following PDPF incorporation, although the individual effects of protein and fibre cannot be isolated from the other compositional changes. In contrast, Gagneten et al. [49] observed a lower maximum fracture force after fibre enrichment of GF cookies, illustrating that fibre incorporation does not invariably increase hardness and that its effect depends on fibre source and the resulting matrix structure.

#### 3.3.3. Colour Analysis

**Figure 3:**
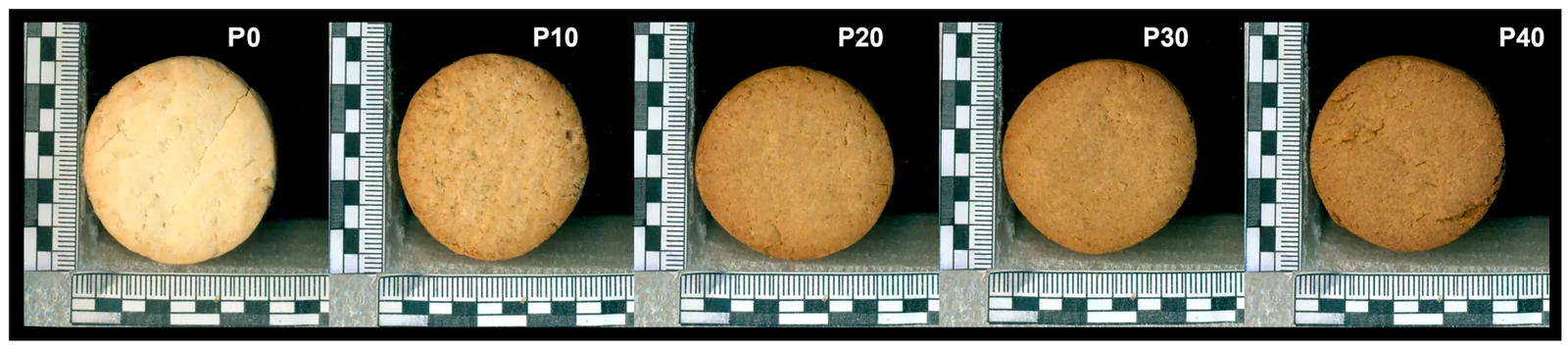
Images of cookies made with rice flour and with different levels of partially defatted pistachio flour (PDPF) substitution. P0: Control cookie made with 100% rice flour. P10, P20, P30, and P40: Cookies made with rice flour and different levels of rice flour substitution with PDPF (10%, 20%, 30%, and 40%, respectively).

The colour characteristics of the cookies are presented in Table 4 and Figure 3. PDPF incorporation significantly affected all colour parameters (*p* < 0.05). Lightness (L*) decreased progressively from 81.7 in P0 to 49.3 in P40, indicating pronounced darkening as the substitution level increased. In parallel, a* increased from 6.0 to 17.3, while hue (h) decreased from 76.1 to 60.5. Since all samples were located in the positive a*–b* quadrant, these changes indicate a progressive shift from yellow towards red hues. The b* coordinate was higher in all PDPF-containing cookies than in P0, although it did not follow a progressive trend with substitution level. Chroma (C*) also increased substantially after PDPF incorporation, from 24.9 in P0 to values between 33.1 and 35.5, indicating a more saturated colour, with only minor differences among the formulations containing 20–40% PDPF. The colour difference (ΔE) relative to P0 increased progressively from 17.0 in P10 to 23.0, 31.2, and 35.0 in P20, P30, and P40, respectively. All values substantially exceeded the ΔE > 5 criterion, which is commonly used to indicate clearly visible colour differences [50,51], even at the lowest substitution level (P10). These findings suggest that substituting rice flour with PDPF results in progressively darker and redder cookies, with higher colour saturation than the control.

The observed changes can be partly attributed to the intrinsic colour of PDPF. Mendoza-Pérez et al. [10] showed that pistachio flours obtained by single-screw pressing exhibit relatively low L* values and pronounced positive a* and b* coordinates, consistent with the darker and more reddish-yellow colour observed as their proportion in the cookies increased. Similar darkening has been reported for other oil-extraction by-products. Pycia et al. [52], for example, observed a significant reduction in L* and increased a* and b* when walnut oilcake was incorporated into wheat bread.

Research has demonstrated that the colour of bakery products is determined not only by the thermal breakdown of natural pigments and lipids in the flour used, but also by non-enzymatic browning reactions, such as Maillard reactions and caramelisation, that take place during the baking process [53,54]. Because PDPF incorporation progressively increased the protein content of the formulations, a greater availability of amino groups may have also contributed to differences in Maillard browning. Therefore, the observed colour changes may reflect the combined effects of the intrinsic colour of PDPF and baking-induced browning, although their relative contributions cannot be quantified because Maillard reaction products were not measured.

### 3.4. Sensory Properties of Cookies

**Figure 4:**
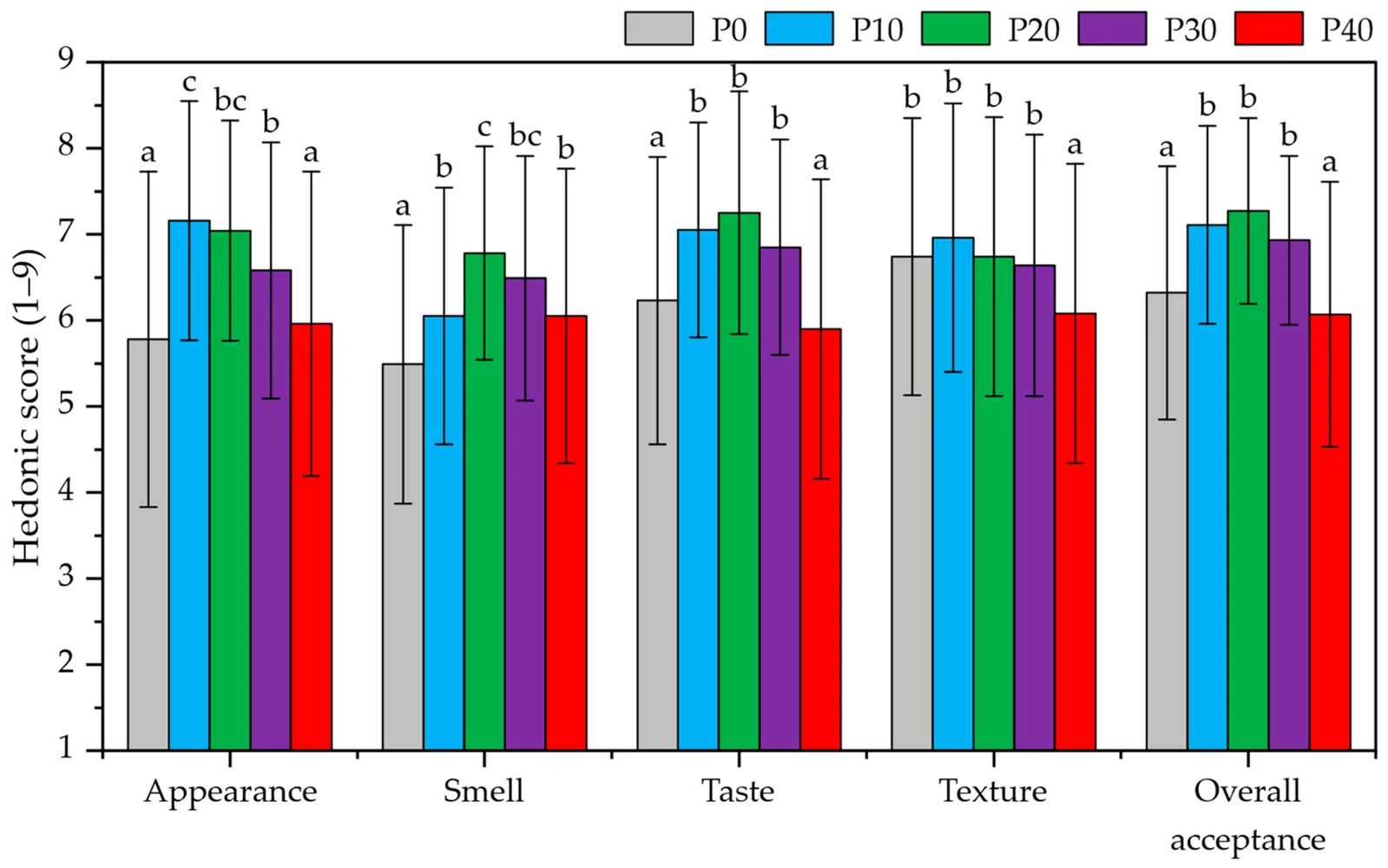
Results of consumer testing of cookies made with rice flour and with different levels of substitution with partially defatted pistachio flour. Bars represent mean values, and error bars indicate standard deviations. Different letters indicate significant differences among formulations within each sensory attribute (*p* < 0.05).

The sensory characteristics of the cookies were evaluated by untrained consumers recruited from the University of Valladolid community (Spain) using a nine-point hedonic scale, considering appearance, smell, texture, taste, and overall acceptance. The results are presented in Figure 4.

Cookie appearance was favourably affected by moderate PDPF incorporation. P10, P20, and P30 received significantly higher appearance scores than both P0 and P40. Among the enriched formulations, P10 obtained the highest score (7.2), although it did not differ significantly from P20 (7.0), whereas P30 showed an intermediate score (6.6). In contrast, P40 (6.0) did not differ from the control (5.8).

Sensory texture liking showed a markedly different pattern from instrumental breaking force. Despite the strong increase in maximum breaking force with increasing PDPF incorporation, no significant differences in texture liking were observed among P0, P10, P20, and P30, with scores ranging from 6.6 to 7.0. Only P40 received a significantly lower texture-liking score (6.1). Therefore, the substantial increase in instrumental breaking force was not accompanied by a significant reduction in texture liking up to 30% PDPF, whereas the highest substitution level resulted in significantly lower texture liking. Frauenhoffer et al. [55] reported that both excessive and insufficient perceived hardness reduced consumer liking of gluten-free cookies. Thus, the increased breaking force observed up to P30 may still have corresponded to a texture within an acceptable range for consumers.

Smell was positively affected by PDPF incorporation. All PDPF-containing cookies received significantly higher scores than P0. P20 obtained the highest score (6.8), although it did not differ significantly from P30 (6.5), whereas P10 and P40 showed lower but still significantly higher values than the control. This response suggests that the aroma associated with PDPF was generally well accepted, particularly at intermediate substitution levels. A similar response was observed for taste. Cookies containing 10–30% PDPF received significantly higher scores (6.9–7.2) than both P0 (6.2) and P40 (6.0), with no significant differences among P10, P20, and P30. Thus, moderate PDPF incorporation enhanced consumer liking of cookie taste, whereas increasing the substitution level to 40% did not maintain this improvement. In the free-response section of the tasting sheet, smell and taste were frequently mentioned by consumers as relevant factors in their evaluations.

Overall acceptance closely reflected the response observed for taste. P10, P20, and P30 achieved the highest scores (7.1, 7.2, and 6.9, respectively), with no significant differences among these formulations, whereas P0 and P40 showed significantly lower and statistically similar scores (6.3 and 6.1, respectively). Therefore, PDPF incorporation at levels between 10% and 30% improved overall consumer acceptance relative to the rice-flour control, while increasing the substitution level to 40% eliminated this sensory advantage.

The existence of an intermediate range of ingredient incorporation providing favourable sensory properties is consistent with previous studies involving seed- and nut-derived ingredients in bakery products. Ganorkar & Jain [56] reported that cookies containing 15% roasted flaxseed flour showed sensory characteristics comparable to those of the control, illustrating that moderate incorporation levels can be well accepted. Similarly, Martínez et al. [20] showed that the sensory response to cookies made with defatted seed flours strongly depended on the botanical source, with flax- and sesame-based formulations receiving more favourable evaluations than those prepared with chia or poppy flour. Ojinnaka & Agubolum [57], who substituted 10%, 20%, 30%, and 40% of wheat flour with cashew nut paste, reported a decline in sensory attributes at the highest substitution level. Pineli et al. [48] also reported that cookies containing 25% partially defatted baru flour achieved acceptance comparable to the wheat-flour control for certain sensory attributes (texture and taste) and in specific consumer groups. These findings highlight the importance of optimising both the type and level of incorporation of seed- and nut-derived ingredients to improve the nutritional profile of bakery products without compromising sensory quality [20].

## 4. Conclusions

This study demonstrates that PDPF can be effectively incorporated into GF cookie formulations as both an ingredient for nutritional enrichment and a structural component capable of modifying dough and product characteristics.

Progressive replacement of rice flour with PDPF shifted the formulation from a starch-rich matrix towards one with higher protein, fibre, and ash contents, while the total fat content was maintained through formulation adjustment. This nutritional improvement was accompanied by marked changes in dough rheology, particularly an increase in compliance and a reduction in resistance to flow as PDPF incorporation increased. The significant relationships observed between composition and creep-recovery parameters indicate that these rheological changes were closely associated with the progressive replacement of rice flour by the protein- and fibre-rich pistachio ingredient, although the simultaneous variation in formulation components prevents their individual contributions from being distinguished.

The structural modifications observed in the unbaked dough were also related to the characteristics developed after baking. Formulations showing greater dough deformability and lower steady-state viscosity subsequently produced harder cookies, highlighting that increased deformability of the raw dough does not necessarily translate into a softer final product. This relationship emphasises the relevance of considering dough rheology together with formulation composition when predicting the technological behaviour of GF bakery systems. PDPF incorporation also reduced cookie spread and progressively modified colour, producing darker and redder products with increasing substitution level. These changes reflect the strong influence of PDPF on both dough structure and the physical appearance of the final product.

From a product-development perspective, PDPF substitution levels between 10% and 30% provided a favourable balance between nutritional enrichment and consumer acceptance despite the marked technological changes. Within this range, the increases in protein and fibre contents were accompanied by favourable appearance, taste, and overall acceptance, while texture liking remained comparable to that of the control despite the pronounced increase in instrumental breaking force. This result is particularly relevant because it shows that substantial changes in instrumental breaking force do not necessarily translate into a significant reduction in consumers’ texture liking at moderate substitution levels. At 40% substitution, nutritional enrichment continued and the physical effects of PDPF became more pronounced, but appearance, taste, and overall consumer liking did not differ significantly from the control, whereas texture liking was significantly lower.

Overall, these findings indicate that maximising PDPF incorporation is not necessarily the most appropriate formulation strategy. Instead, an intermediate substitution range appears more suitable for achieving a compromise between improved nutritional quality and acceptable technological and sensory properties. Among the formulations tested, P30 offered the most favourable balance when prioritising nutritional enrichment, as it provided the highest PDPF incorporation while maintaining improved sensory acceptance compared with the control. The study therefore supports the use of PDPF as a promising high-value ingredient for the development of nutritionally improved GF bakery products, while also enabling the valorisation of pistachio oil-extraction by-products. Further studies could explore the digestibility and bioaccessibility of nutrients and bioactive compounds in PDPF-enriched cookies, extend sensory evaluation to broader consumer populations, and assess the application of this ingredient in other gluten-free bakery products.

## Figures and Tables

**Table 1:** Formulation of cookies made with rice flour and different levels of partially defatted pistachio flour (PDPF) substitution.

Ingredient	Control	PDPF Substitution Level			
	P0	P10	P20	P30	P40
Rice flour	50.00	44.90	39.90	34.80	29.80
PDPF	0.00	5.50	10.90	16.30	21.70
Butter	23.00	22.10	21.20	20.30	19.40
Sugar	15.00	15.00	15.00	15.00	15.00
Water	11.00	11.50	12.00	12.60	13.10
Sodium bicarbonate	0.50	0.50	0.50	0.50	0.50
Vanilla extract	0.25	0.25	0.25	0.25	0.25
Salt	0.25	0.25	0.25	0.25	0.25

P0: Control cookie made with 100% rice flour. P10, P20, P30, and P40: Cookies prepared at nominal rice flour substitution levels of 10%, 20%, 30%, and 40%, respectively, before formulation adjustments. Ingredient quantities are expressed as g/100 g of dough. The moisture contents of rice flour, PDPF, and butter were 12.5%, 3.8%, and 16.0%, respectively.

**Table 2:** Rheological properties of doughs made with rice flour and different levels of partially defatted pistachio flour (PDPF) substitution.

Parameters	Control	PDPF Substitution Level				SE
	P0	P10	P20	P30	P40	
Strain sweeps						
τ_max_ (kPa)	0.22 b	0.22 b	0.17 a	0.19 ab	0.21 b	0.02
Crossover stress (kPa)	1.8 a	1.8 a	1.7 a	1.8 a	1.7 a	0.1
Frequency sweeps						
G_1_′ (kPa)	163 b	155 b	118 a	117 a	121 a	5
*a*	0.126 a	0.133 a	0.142 b	0.147 b	0.149 b	0.003
G_1_″ (kPa)	33 b	34 b	27 a	27 a	29 a	1
*b*	0.248 ab	0.242 a	0.247 ab	0.255 b	0.248 ab	0.003
tan(δ)_1_	0.203 a	0.219 b	0.230 bc	0.236 c	0.247 d	0.002
*c*	0.122 b	0.109 a	0.105 a	0.109 a	0.100 a	0.004
Creep-recovery test						
J_0c_ (MPa^−1^)	7.5 a	7.5 a	8.6 ab	9.7 b	11.8 c	0.5
J_1c_ (MPa^−1^)	5.9 a	6.8 ab	7.3 b	9.1 c	10.6 cd	0.4
λ_1c_ (s)	3.8 a	3.8 a	3.7 a	3.3 a	3.5 a	0.4
µ_0_ (MPa·s)	7.6 c	6.6 bc	6.4 bc	5.3 ab	4.2 a	0.9
R^2^ creep	0.995	0.995	0.994	0.993	0.995	0.001
J_max_ (MPa^−1^)	21 a	23 ab	28 b	30 bc	36 c	2
[(J_0c_ + J_1c_)/J_max_] (%)	63 a	62 a	58 a	62 a	62 a	5
J_0r_ (MPa^−1^)	8.4 a	8.6 a	10.4 ab	11.6 b	13.4 c	0.6
J_1r_ (MPa^−1^)	8.3 a	9.8 ab	11.9 bc	12.4 c	14.5 d	0.9
λ_1r_ (s)	23 a	27 ab	28 b	24 ab	23 a	1
R^2^ recovery	0.955	0.970	0.956	0.953	0.953	0.006

P0: Control dough made with 100% rice flour. P10, P20, P30, and P40: Doughs made with rice flour and different levels of rice flour substitution with partially defatted pistachio flour (10%, 20%, 30%, and 40%, respectively). τ_max_: maximum stress that the samples can withstand (end of the linear viscoelastic region). Crossover stress: the stress at which G′ = G″. The power law model was fitted to the experimental frequency sweep data (G′ = G_1_′⋅ω^a^; G″ = G_1_″⋅ω^b^; tanδ = (tanδ)_1_⋅ω^c^). G_1_′, G_1_″ and tan(δ)_1_: represent the elastic modulus, viscous modulus, and loss tangent at a frequency of 1 Hz, respectively. *a*, *b*, and *c*: exponents quantifying the degree of dependence of G′, G″, and tanδ with the oscillation frequency. The subscript “c” refers to the creep phase and the subscript “r” to the recovery phase. J_0_c and J_0_r: instantaneous compliances. J_1_c and J_1_r: retarded compliances. J_max_: maximum creep compliance at the end of the creep step. λ_1c_ and λ_1r_: retardation time. μ_0_: steady-state viscosity. R^2^: coefficient of determination. SE: pooled standard error obtained from ANOVA. Values with different letters in each row indicate significant differences between means (*p* < 0.05).

**Table 3:** Proximate composition of cookies made with rice flour and different levels of partially defatted pistachio flour (PDPF) substitution.

Parameters	Control	PDPF Substitution Level				SE
	P0	P10	P20	P30	P40	
Energy (kcal/100 g)	509.04	503.21	499.98	497.54	493.27	-
Moisture (g/100 g)	0.6 a	1.2 b	1.4 b	1.3 b	1.6 b	0.2
Carbohydrates (g/100 g)	71 e	67 d	63 c	59 b	55 a	1
Fibre (g/100 g)	0.8 a	1.5 ab	2.2 bc	3.0 cd	3.7 d	0.3
Fat (g/100 g)	23.1 a	23.0 a	23.0 a	23.1 a	23.0 a	0.9
Protein (g/100 g)	4.1 a	6.6 b	9.2 c	11.7 d	14.3 e	0.3
Ash (g/100 g)	0.60 a	0.94 b	1.29 c	1.63 d	1.98 e	0.08

P0: Control cookie made with 100% rice flour. P10, P20, P30, and P40: Cookies made with rice flour and different levels of rice flour substitution with partially defatted pistachio flour (10%, 20%, 30%, and 40%, respectively). SE: pooled standard error obtained from ANOVA. Values with different letters in each row indicate significant differences between means (*p* < 0.05).

**Table 4:** Physical parameters of cookies made with rice flour and different levels of partially defatted pistachio flour (PDPF) substitution.

Parameters	Control	PDPF Substitution Level				SE
	P0	P10	P20	P30	P40	
Morphogeometry						
Diameter (mm)	38.9 a	39.2 a	39.2 a	39.0 a	39.0 a	0.2
Thickness (mm)	7.7 a	8.4 c	8.3 bc	8.2 b	8.2 bc	0.1
Spread factor	5.1 b	4.7 a	4.7 a	4.8 a	4.7 a	0.1
Texture						
Maximum force (N)	5.0 a	6.6 b	11.1 c	22.1 d	23.8 e	0.5
Colour						
L*	81.7 e	67.6 d	62.1 c	53.3 b	49.3 a	0.5
a*	6.0 a	12.9 b	14.5 c	16.5 d	17.3 e	0.1
b*	24.2 a	30.6 b	32.4 d	31.6 c	30.6 b	0.3
h	76.1 e	67.1 d	65.9 c	62.4 b	60.5 a	0.4
C*	24.9 a	33.1 b	35.5 c	35.5 c	35.4 c	0.3
∆E	-	17.0 a	23.0 b	31.2 c	35.0 d	0.5

P0: Control cookie made with 100% rice flour. P10, P20, P30, and P40: Cookies made with rice flour and different levels of rice flour substitution with partially defatted pistachio flour (10%, 20%, 30%, and 40%, respectively). L*: lightness; a* and b*: colour coordinates; C*: chroma; h: hue; ∆E: colour difference with respect to P0. SE: pooled standard error obtained from ANOVA. Values with different letters in each row indicate significant differences between means (*p* < 0.05).

## Data Availability

The raw data supporting the conclusions of this article will be made available by the authors on request.

## References

[1] Hadidi M., Aghababaei F., Gonzalez-Serrano D.J., Goksen G., Trif M., McClements D.J., Moreno A. (2024). Plant-Based Proteins from Agro-Industrial Waste and by-Products: Towards a More Circular Economy. Int. J. Biol. Macromol..

[2] Jasińska-Kuligowska I., Kuligowski M., Wyszyński M., Kidoń M. (2025). Upcycling of Sunflower and Sesame Press Cakes as Functional Ingredients in Cookies. Sustainability.

[3] Hadidi M., Garcia S.R., Ziogkas D., McClements D.J., Moreno A. (2024). Cereal Bran Proteins: Recent Advances in Extraction, Properties, and Applications. Crit. Rev. Food Sci. Nutr..

[4] Zhang M., Wang O., Cai S., Zhao L., Zhao L. (2023). Composition, Functional Properties, Health Benefits and Applications of Oilseed Proteins: A Systematic Review. Food Res. Int..

[5] Gonçalves B., Pinto T., Aires A., Morais M.C., Bacelar E., Anjos R., Ferreira-Cardoso J., Oliveira I., Vilela A., Cosme F. (2023). Composition of Nuts and Their Potential Health Benefits—An Overview. Foods.

[6] FAOSTAT Crops and Livestock Products. https://www.fao.org/faostat/en/#data/QCL.

[7] Guardianelli L., Puppo M.C., Salinas M.V. (2021). Influence of Pistachio By-Product from Edible Oil Industry on Rheological, Hydration, and Thermal Properties of Wheat Dough. LWT.

[8] Rabadán A., Álvarez-Ortí M., Martínez E., Pardo-Giménez A., Zied D.C., Pardo J.E. (2021). Effect of Replacing Traditional Ingredients for Oils and Flours from Nuts and Seeds on the Characteristics and Consumer Preferences of Lamb Meat Burgers. LWT.

[9] Toghiani J., Fallah N., Nasernejad B., Mahboubi A., Taherzadeh M.J., Afsham N. (2023). Sustainable Pistachio Dehulling Waste Management and Its Valorization Approaches: A Review. Curr. Pollut. Rep..

[10] Mendoza-Pérez R.J., Álvarez-Olmedo E., Vicente A., Ronda F., Caballero P.A. (2025). Oil Extraction Systems Influence the Techno-Functional and Nutritional Properties of Pistachio Processing By-Products. Foods.

[11] Sá A.G.A., Moreno Y.M.F., Carciofi B.A.M. (2020). Plant Proteins as High-Quality Nutritional Source for Human Diet. Trends Food Sci. Technol..

[12] Roncero J.M., Álvarez-Ortí M., Pardo-Giménez A., Rabadán A., Pardo J.E., Señoráns F.J. (2021). Influence of Pressure Extraction Systems on the Performance, Quality and Composition of Virgin Almond Oil and Defatted Flours. Foods.

[13] Salinas M.V., Guardianelli L.M., Sciammaro L., Picariello G., Mamone G., Puppo M.C. (2021). Nutritional Ingredient By-Product of the Pistachio Oil Industry: Physicochemical Characterization. J. Food Sci. Technol..

[14] Ramachandran S., Singh S.K., Larroche C., Soccol C.R., Pandey A. (2007). Oil Cakes and Their Biotechnological Applications—A Review. Bioresour. Technol..

[15] Mendoza-Pérez R.J., Náthia-Neves G., Blanco B., Vela A.J., Caballero P.A., Ronda F. (2024). Physicochemical Characterisation of Seeds, Oil and Defatted Cake of Three Hempseed Varieties Cultivated in Spain. Foods.

[16] Radočaj O., Dimić E., Tsao R. (2014). Effects of Hemp (*Cannabis sativa* L.) Seed Oil Press-Cake and Decaffeinated Green Tea Leaves (*Camellia sinensis*) on Functional Characteristics of Gluten-Free Crackers. J. Food Sci..

[17] Prakash K., Naik S.N., Vadivel D., Hariprasad P., Gandhi D., Saravanadevi S. (2018). Utilization of Defatted Sesame Cake in Enhancing the Nutritional and Functional Characteristics of Biscuits. J. Food Process. Preserv..

[18] Suleman D., Bashir S., Hassan Shah F.U., Ikram A., Zia Shahid M., Tufail T., Ahmad Khan A., Ahsan F., Ambreen S., Raza A. (2023). Nutritional and Functional Properties of Cookies Enriched with Defatted Peanut Cake Flour. Cogent Food Agric..

[19] Martendal L.F., Silva M.L.T., da Silva Simão R., Block J.M., de Moraes J.O., Geraldo A.P.G., Feltes M.M.C. (2023). Formulation of Nutritious Gluten-free Cookies with Upcycled Pecan Nut Cake Flour. JSFA Rep..

[20] Martínez E., García-Martínez R., Álvarez-Ortí M., Rabadán A., Pardo-Giménez A., Pardo J.E. (2021). Elaboration of Gluten-Free Cookies with Defatted Seed Flours: Effects on Technological, Nutritional, and Consumer Aspects. Foods.

[21] Vicente A., Villanueva M., Caballero P.A., Lazaridou A., Biliaderis C.G., Ronda F. (2024). Microwave Treatment Enhances the Physical and Sensory Quality of Quinoa-Enriched Gluten-Free Bread. Food Hydrocoll..

[22] Nevara G.A., Giwa Ibrahim S., Syed Muhammad S.K., Zawawi N., Mustapha N.A., Karim R. (2023). Oilseed Meals into Foods: An Approach for the Valorization of Oilseed by-Products. Crit. Rev. Food Sci. Nutr..

[23] AACC International (2010). Approved Methods of Analysis.

[24] Latimer G.W. (2023). Official Methods of Analysis of AOAC international.

[25] Hu H., Wang Y., Shen M., Huang Y., Li C., Nie S., Xie M. (2022). Effects of Baking Factors and Recipes on the Quality of Butter Cookies and the Formation of Advanced Glycation End Products (AGEs) and 5-Hydroxymethylfurfural (HMF). Curr. Res. Food Sci..

[26] Ronda F., Pérez-Quirce S., Villanueva M., Ahmed J., Ptaszek P., Basu S. (2017). Rheological Properties of Gluten-Free Bread Doughs: Relationship With Bread Quality. Advances in Food Rheology and Its Applications.

[27] Ronda F., Villanueva M., Collar C. (2014). Influence of Acidification on Dough Viscoelasticity of Gluten-Free Rice Starch-Based Dough Matrices Enriched with Exogenous Protein. LWT.

[28] Villanueva M., Pérez-Quirce S., Collar C., Ronda F. (2018). Impact of Acidification and Protein Fortification on Rheological and Thermal Properties of Wheat, Corn, Potato and Tapioca Starch-Based Gluten-Free Bread Doughs. LWT.

[29] European Parliament, Council of the European Union (2011). Regulation (EU) No 1169/2011 of the European Parliament and of the Council of 25 October 2011 on the Provision of Food Information to Consumers. Off. J. Eur. Union.

[30] Pareyt B., Talhaoui F., Kerckhofs G., Brijs K., Goesaert H., Wevers M., Delcour J.A. (2009). The Role of Sugar and Fat in Sugar-Snap Cookies: Structural and Textural Properties. J. Food Eng..

[31] Witczak M., Chmielewska A., Ziobro R., Korus J., Juszczak L. (2021). Rapeseed Protein as a Novel Ingredient of Gluten-Free Dough: Rheological and Thermal Properties. Food Hydrocoll..

[32] Villanueva M., Abebe W., Collar C., Ronda F. (2021). Tef [*Eragrostis tef* (*Zucc*.) *Trotter*] Variety Determines Viscoelastic and Thermal Properties of Gluten-Free Dough and Bread Quality. LWT.

[33] Ronda F., Pérez-Quirce S., Angioloni A., Collar C. (2013). Impact of Viscous Dietary Fibres on the Viscoelastic Behaviour of Gluten-Free Formulated Rice Doughs: A Fundamental and Empirical Rheological Approach. Food Hydrocoll..

[34] Vicente A., Villanueva M., Caballero P.A., Lazaridou A., Biliaderis C.G., Ronda F. (2024). Flours from Microwave-Treated Buckwheat Grains Improve the Physical Properties and Nutritional Quality of Gluten-Free Bread. Food Hydrocoll..

[35] Pycia K., Juszczak L. (2022). The Effect of the Addition of Hazelnut or Walnut Flour on the Rheological Characteristics of Wheat Dough. Materials.

[36] Burbano J., Cabezas D., Correa M. (2022). Effect of Walnut Flour Addition on Rheological, Thermal and Microstructural Properties of a Gluten Free-Batter. LWT.

[37] Sivaramakrishnan H., Senge B., Chattopadhyay P. (2004). Rheological Properties of Rice Dough for Making Rice Bread. J. Food Eng..

[38] Vela A.J., Villanueva M., Ronda F. (2021). Low-Frequency Ultrasonication Modulates the Impact of Annealing on Physicochemical and Functional Properties of Rice Flour. Food Hydrocoll..

[39] Vela A.J., Villanueva M., Ronda F. (2023). Physical Modification Caused by Acoustic Cavitation Improves Rice Flour Bread-Making Performance. LWT.

[40] Ahmad S., Naz A., Usman M., Amjad A., Pasha I., Farooq U. (2022). Impediment Effect of Chemical Agents (Additives) on Gluten Development in Cookie Dough. J. Food Sci. Technol..

[41] Xu J., Zhang Y., Wang W., Li Y. (2020). Advanced Properties of Gluten-Free Cookies, Cakes, and Crackers: A Review. Trends Food Sci. Technol..

[42] Mancebo C.M., Rodríguez P., Martínez M.M., Gómez M. (2018). Effect of the Addition of Soluble (Nutriose, Inulin and Polydextrose) and Insoluble (Bamboo, Potato and Pea) Fibres on the Quality of Sugar-snap Cookies. Int. J. Food Sci. Technol..

[43] Chung H.J., Cho A., Lim S.T. (2014). Utilization of Germinated and Heat-Moisture Treated Brown Rices in Sugar-Snap Cookies. LWT Food Sci. Technol..

[44] Dapčević Hadnađev T.R., Torbica A.M., Hadnađev M.S. (2013). Influence of Buckwheat Flour and Carboxymethyl Cellulose on Rheological Behaviour and Baking Performance of Gluten-Free Cookie Dough. Food Bioproc. Tech..

[45] Sarabhai S., Prabhasankar P. (2015). Influence of Whey Protein Concentrate and Potato Starch on Rheological Properties and Baking Performance of Indian Water Chestnut Flour Based Gluten Free Cookie Dough. LWT Food Sci. Technol..

[46] Altındağ G., Certel M., Erem F., İlknur Konak Ü. (2015). Quality Characteristics of Gluten-Free Cookies Made of Buckwheat, Corn, and Rice Flour with/without Transglutaminase. Food Sci. Technol. Int..

[47] Laguna L., Sanz T., Sahi S., Fiszman S.M. (2014). Role of Fibre Morphology in Some Quality Features of Fibre-Enriched Biscuits. Int. J. Food Prop..

[48] de Oliveira Pineli L.D.L., de Carvalho M.V., de Aguiar L.A., de Oliveira G.T., Celestino S.M.C., Botelho R.B.A., Chiarello M.D. (2015). Use of Baru (Brazilian Almond) Waste from Physical Extraction of Oil to Produce Flour and Cookies. LWT Food Sci. Technol..

[49] Gagneten M., Pieniazek F., Archaina D., Messina V., Salvatori D., Schebor C. (2023). Texture Study of Gluten-Free Cookies Added with Fibre by Instrumental, Image and Sensory Analysis. J. Food Meas. Charact..

[50] Abebe W., Ronda F. (2014). Rheological and Textural Properties of Tef [*Eragrostis tef* (Zucc.) Trotter] Grain Flour Gels. J. Cereal Sci..

[51] García-Viguera C., Zafrilla P., Ames J.M., Hofmann T.F. (2001). Changes in Anthocyanins during Food Processing: Influence on Color. Chemistry and Physiology of Selected Food Colorants.

[52] Pycia K., Kapusta I., Jaworska G. (2020). Walnut Oil and Oilcake Affect Selected the Physicochemical and Antioxidant Properties of Wheat Bread Enriched with Them. J. Food Process. Preserv..

[53] Ameur L., Mathieu O., Lalanne V., Trystram G., Birlouezaragon I. (2007). Comparison of the Effects of Sucrose and Hexose on Furfural Formation and Browning in Cookies Baked at Different Temperatures. Food Chem..

[54] Tarek-Tilistyák J., Agócs J., Lukács M., Dobró-Tóth M., Juhász-Román M., Dinya Z., Jekő J., Máthé E. (2014). Novel Breads Fortified through Oilseed and Nut Cakes. Acta Aliment..

[55] Frauenhoffer S., Ola E., Jervis S., Gauch S.E., Seo H. (2025). Comparison Analyses of Consumer Acceptance, Evoked Emotions, and Purchase Intent Between Gluten-Containing Versus Gluten-Free Chocolate Chip Cookie Products. J. Food Sci..

[56] Ganorkar P.M., Jain R.K. (2014). Effect of Flaxseed Incorporation on Physical, Sensorial, Textural and Chemical Attributes of Cookies. Int. Food Res. J..

[57] Ojinnaka M., Agubolum F. (2013). Nutritional and Sensory Properties of Cashew Nut-Wheat Based Cookies. Am. J. Food Nutr..

